# In Vivo Assessment of Skin Surface Pattern: Exploring Its Potential as an Indicator of Bone Biomechanical Properties

**DOI:** 10.3390/bioengineering10121338

**Published:** 2023-11-21

**Authors:** Jean-Charles Aurégan, Catherine Bosser, Manon Bachy-Razzouk, Morad Bensidhoum, Thierry Hoc

**Affiliations:** 1 B3OA, UMR CNRS 7052, Inserm U1271 Université de Paris, 10 avenue de Verdun, 75010 Paris, France; jean-charles.auregan@aphp.fr (J.-C.A.); manon.bachy@aphp.fr (M.B.-R.); morad.bensidhoum@cnrs.fr (M.B.); 2 Orthopedics Department, Université Paris-Saclay, AP-HP, Hôpital Antoine Béclère, 157, Rue de la Porte-de-Trivaux, 92140 Clamart, France; 3 HealthDataSciences, 45, Chemin du Barthélémy, 69260 Charbonnières-les-Bains, France; 4 Orthopedics Department, Sorbonne Université, AP-HP, Hôpital Trousseau, 26, Avenue du Docteur-Arnold-Netter, 75012 Paris, France; 5 Mechanical Department, École Centrale de Lyon, MSGMGC, 36, Avenue Guy-de-Collongue, 69134 Ecully, France

**Keywords:** bone mechanics, bone aging, osteogenesis imperfecta, skin surface, collagen

## Abstract

The mechanical properties of bone tissue are the result of a complex process involving collagen–crystal interactions. The mineral density of the bone tissue is correlated with bone strength, whereas the characteristics of collagen are often associated with the ductility and toughness of the bone. From a clinical perspective, bone mineral density alone does not satisfactorily explain skeletal fragility. However, reliable in vivo markers of collagen quality that can be easily used in clinical practice are not available. Hence, the objective of the present study is to examine the relationship between skin surface morphology and changes in the mechanical properties of the bone. An experimental study was conducted on healthy children (n = 11), children with osteogenesis imperfecta (n = 13), and women over 60 years of age (n = 22). For each patient, the skin characteristic length (SCL) of the forearm skin surface was measured. The SCL quantifies the geometric patterns formed by wrinkles on the skin’s surface, both in terms of size and elongation. The greater the SCL, the more deficient was the organic collagen matrix. In addition, the bone volume fraction and mechanical properties of the explanted femoral head were determined for the elderly female group. The mean SCL values of the healthy children group were significantly lower than those of the elderly women and osteogenesis imperfecta groups. For the aged women group, no significant differences were indicated in the elastic mechanical parameters, whereas bone toughness and ductility decreased significantly as the SCL increased. In conclusion, in bone collagen pathology or bone aging, the SCL is significantly impaired. This in vivo skin surface parameter can be a non-invasive tool to improve the estimation of bone matrix quality and to identify subjects at high risk of bone fracture.

## 1. Introduction

Bone tissue is a complex, multiscale composite material that continuously evolves to adapt to its environment, particularly mechanically, through bone remodeling [1,2]. Based mainly on three types of cells, osteoclasts, osteoblasts, and osteocytes, this remodeling process results in a subtle arrangement of collagen and intrinsically bound minerals, which endows bone tissue with exceptional mechanical behavior [3,4,5]. In particular, mineral content is a major determinant of its strength, which can be measured in vivo using dual-energy X-ray absorptiometry (DEXA) in certain areas of the skeleton to identify osteoporotic patients and determine the risk of bone fracture [6,7]. However, since the early 2000s, many studies have shown that mineral content alone does not satisfactorily explain skeletal fragility and that bone remodeling parameters must be considered in clinical practice [8,9,10].

Therefore, over the past 20 years, researchers have primarily focused on the crystal and collagen quality of bone tissue [11,12,13,14]. Additionally, the effects of patient age and various pathologies have been investigated [15,16,17,18,19]. Recent studies have benefited from the development of new experimental laboratory techniques for material characterization, thus allowing more samples to be analyzed while achieving unprecedented experimental resolutions [20,21,22,23,24,25].

Additionally, the exponential growth of computing capacities has resulted in the emergence of approaches for molecular or ab initio dynamics simulations of various bone components at the nanometric scale and their interactions [26,27]. Even though this field is currently being actively investigated, several consensuses have emerged that highlight the effect of the collagen structure on the quality of the resulting crystal and its direct association with specific mechanical properties. Studies have shown that the mechanism of fracture cannot be reduced to knowledge regarding the strength of bone tissue [9]. Toughness and ultimate strain (the ability of a material to deform without breaking) are important indicators that are typically associated with the quality of the bone matrix [11,16,28]. Bone toughness is an intrinsic property of the material and is defined as the amount of energy per unit volume that the bone can absorb before fracturing. Bone fracture depends on toughness, which characterizes the quality of the bone tissue, but also on other factors such as the patient’s anatomy or the nature of the trauma. In particular, the structural integrity of the collagen network and the accumulation of age-related glycation end products (AGEs), which is a family of the non-enzymatic type of collagen crosslinks, play a significant role in bone toughness [15,29,30]. AGEs are a type of collagen cross-link that forms mainly in the presence of sugar and are non-enzymatic, as they form during protein glycation in the helical regions between tropocollagen molecules.

Consequently, the consideration of fracture risk by DEXA has been enriched by new computational techniques that consider both the microarchitecture of the trabecular bone (trabecular bone score [TBS]) [31] and the equivalent strain within the bone (bone strain index [BSI]). The BSI is derived from finite element simulation, which is the gold standard tool for engineering structure design. The underlying assumption of the BSI is that an increase in fracture risk is proportional to an increase in strain. Using this approach, bone fragility is no longer based solely on mineral content or geometry but incorporates the quality of the bone matrix [32]. Although the BSI is a new index, the first results are promising and confirm, as suggested by Hart in 2017 [33], the necessity to consider the organic phase of the bone, which is typically not considered in clinical investigations.

**Figure 1:**
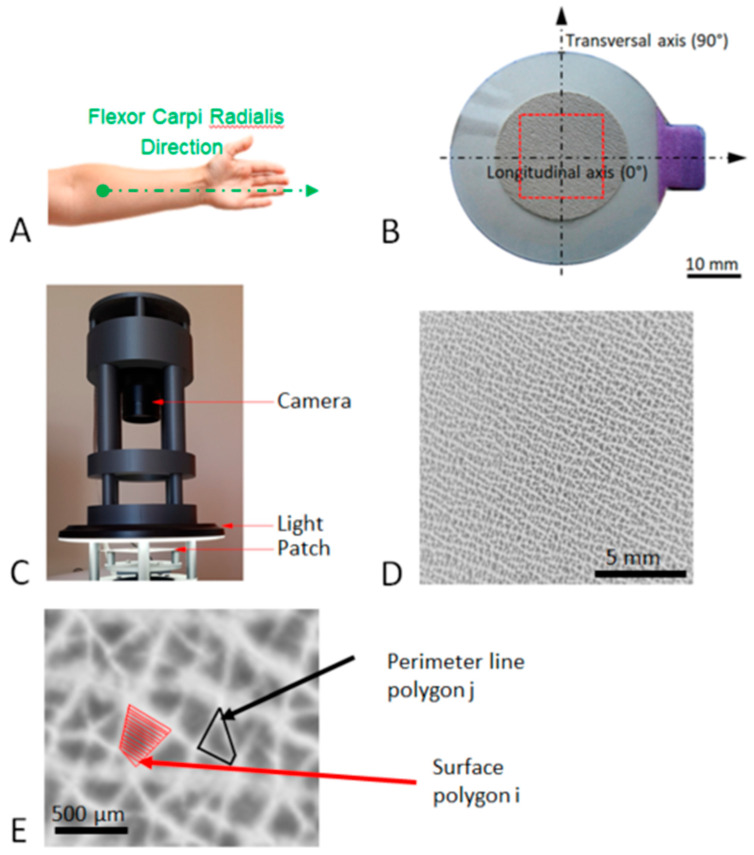
Image acquisition process of the patch. (**A**) Anatomical location of the patch. (**B**) Representation of the patch used in the present study: the internal diameter of the patch is 24 mm, the red zone corresponds to the observation area (15 mm × 15 mm), and the longitudinal axis is oriented toward the relief of the flexor carpi radialis tendon. (**C**) Acquisition device using a circular LED light and a 12-million-pixel camera. (**D**) Typical high-resolution image of the patch analysis area. (**E**) Enlargement of image (**D**) showing the perimeter as the length of the line around a polygon and the area of a polygon at the skin surface.

If collagen (organic matter) is a key factor in skeletal ductility and toughness, then reliable biomarkers that can be easily used in clinical practice are required. In this context, Shuster [34,35] proposed a theory of cross-aging between bone and skin by assuming that the collagen in these two connective tissues ages at the same rate. In fact, the degradation of structural [36,37], mechanical [38], textural [39], functional, and physiological [40] aspects of the skin are now well documented between young and elderly populations, and some of the manifestations reported are similar to those of bone aging. All these changes in the skin are mainly due to the modification of the quality of the dermis with age [41], which results in an increase in the density of elastin fibers (recoil) and a decrease in the density of collagen fibres (strength). This elastin/collagen ratio can be determined by measuring the SCL, which describes the morphology of the polygonal network made up of wrinkles (see Figure 1) observed on the skin surface [42].

The aim of this study is to investigate the ability of microrelief of the skin surface, described by a characteristic length called the SCL, to assess a change in the mechanical properties of bone tissue. The underlying scientific hypothesis is that a change in the quality of the dermis is associated with a change in the quality of the organic bone matrix. From a clinical perspective, a non-invasive test that can enhance the diagnosis of bone fractures via DEXA is desirable. Two clinical studies were conducted to demonstrate whether or not SCL can detect changes in bone tissue. The first study compared the SCL of 11 non-pathological children and 13 patients with osteogenesis imperfecta. Osteogenesis imperfecta is a constitutive bone pathology caused by a mutation in collagen, which results in a significant decrease in bone toughness and ductility [43,44]. The second study was conducted on 22 women over the age of 60 years who suffered a femoral neck fracture after a fall from their height, a population considered to have more fragile characteristics than the general population. In particular, the toughness and ductility of the trabecular bone taken from the femoral head were analyzed in relation to the SCL value.

## 2. Materials and Methods

### 2.1. Osteogenesis Imperfecta and Non-Pathological Population

To analyze the effects of osteogenesis imperfecta, two groups were analyzed in the present study. The first group consisted of 11 healthy children with no known pathology, referred to as “non-pathological”. The second group was composed of 13 children with mild osteogenesis imperfecta, denoted as “OI”. All OI patients were consecutively managed in the orthopedic surgery department of Armand Trousseau Hospital, France. The present study was conducted in accordance with the Declaration of Helsinki and approved by the South Mediterranean IV Ethics Committee (reference no.: 2020-A01416-33). Children’s assets have been obtained, and all the patients and parents of included children provided written informed consent before participating in the study. The different characteristics of patients are given in Table 1. All patients were anonymized by Dr. M. Bachy prior to the measurement of the various parameters in accordance with current French law. A double-blind procedure was used to process the data.

The mean height and weight of the healthy group were not significantly different from the mean values derived from the growth and weight curves of the French population calculated for this group, i.e., 1.68 ± 0.1 m and 54 ± 8.5 kg, respectively. By contrast, the mean height and weight of the OI group were significantly lower (*p* = 0.008 and *p* = 0.05, respectively) than the mean values derived from the growth and weight curves of the French population calculated for this group, respectively, i.e., 1.56 ± 0.1 m and 48.2 ± 9.8 kg, respectively

### 2.2. Senior Women Population

To analyze the effect of aging, a group of 22 women over the age of 60 was studied. These women suffered a hip fracture after a simple fall and were referred to as “senior women”. A simple fall for an older person is a fall such as tripping, slipping, or losing balance, as opposed to a fall with a high-energy impact such as a road traffic accident. All patients were consecutively managed in the orthopedic surgery department of the Antoine Béclère Hospital, France. Excluded from this group were patients with a medical history, treatments, habits competing with metabolism (long-term corticosteroids, smoking, alcohol consumption > 3 units/day), or bone or skin pathology [45]. The present study was conducted in accordance with the Declaration of Helsinki and approved by the South Paris Ethics Committee (reference no.: PP-14-018). All the patients provided written informed consent before participating in the study. The different characteristics of senior women are given in Table 2. All patients were anonymized by Dr. Auregan prior to the measurement of the various parameters, in accordance with current French law. A double-blind procedure was used to process the data.

### 2.3. Skin Characteristic Length (SCL)

Fine grooves and lines of different depths were observed on the skin surface, which segregated the skin surface into irregular geometric shapes, thus resulting in a network of polygons that evolve with age and depend on the dermis quality. To accurately reproduce this network on the skin surface and automatically quantify the area and shape of the polygons, a technique based on silicone replicas (SILFLO^®^, silicone polymer, and Monaderm catalyst, Monaco) and employed in the cosmetic industry for years [46,47] was used. In the present study, a skin imprint, referred to as “patch” hereafter, was performed on the patient’s forearm, which was assumed to be an area not exposed to the sun. The patient’s forearm was cleaned and positioned such that its anterior aspect faced upward. An adhesive crown was applied 5 cm from the elbow, with the landmark placed toward the relief of the flexor carpi radialis tendon (Figure 1A). A specific device (Figure 1C), including a circular LED light (external diameter 16 cm, white light 3500 K) and a 12-million-pixel camera (Raspberry Pi HQ) mounted on a three-dimensionally printed support, was used to acquire the skin patch image. The area in the center of the patch (15 mm × 15 mm) was selected (Figure 1D) and converted into an 8-bit grayscale image. The detailed procedure is described in a previous paper [42]. In summary, as a skin replica, the lowest points correspond to the basins, which are the skin polygons (black = 0). The highest points correspond to the wrinkle (white = 255). The image segmentation was processed using a Marker-Controlled Watershed algorithm. This algorithm treats pixel values as local topography and flood basins from markers. The success of this type of transformation depends on the choice of the markers. Mathematical morphology was used to compute the extended regional minima after an H-minima transformation in a custom Python routine (v3.7, Python Software Foundation, Wilmington, DE, USA). This method provides access to the area (*A_i_*) and perimeter (*P_i_*) of each polygon *i*, given in Figure 1E. The mean value of SCL for each skin surface derived from the equation provided by the paper [42] follows:

(1) $$SCL=\frac{1}{Nb}\sum_{i=1}^{Nb}\left[\sqrt{A_{i}}+\frac{P_{i}}{4}\right],$$

where *N_b_* denotes the number of polygons in the field of 15 × 15 mm; in this formulation, polygons are considered as rectangles, and $\sqrt{A_{i}}$ corresponds to the side length of the equivalent area square. *P_i_*/4 corresponds to a length representing a quarter of the perimeter. Here, the SCL considers both the size and geometric aspects of the polygons. Based on six images of the same patch taken at different times, the measurement error is less than 0.5% of the SCL value.

### 2.4. Ratio of Elastin to Collagen in the Dermis of Senior Women (REC)

For the twenty-two elderly women, a 5 × 5 mm^2^ skin biopsy was retrieved in the gluteal region and stored at −20 °C. The upper dermis of the collected skin biopsy was imaged using two-photon confocal imaging (A1RMP PLUS^®^, Nikon, Tokyo, Japan). The excitation wavelength was 900 nm. Two channels with specific bandpass filters of 400–490 and 500–550 nm were used to collect second harmonic generation (SHG) light from collagen and autofluorescence (AF) light from elastin, respectively. A 25×, 1.1-NA water immersion objective (CFI Apo LWD 25XW, Nikon, Tokyo, Japan) was used for imaging. The field of view was 512 × 512 µm^2^. The resolution was 0.5 µm/pixel. A custom routine was developed to compute the elastin-to-collagen ratio (REC) for each sample as the number of elastin pixels over the number of collagen pixels in the entire image stack. A k-means clustering method was used to determine an automatic grey-level threshold to separate the elastin signal from the collagen. All detailed description of the method can be found in Bachy et al. [42]

### 2.5. Bone Volume Fraction of Trabecular Bone of Senior Women

For the senior group, the femoral head was harvested and immediately stored at −20 °C for up to one month prior to mechanical testing. Then, a trabecular bone sample of cylindrical shape was prepared using a water bath trephine at a standardized location and direction. Both end surfaces were polished (#1200 grit) to obtain fresh standard specimens with an average radius of 3.5 mm and an average length of 8.7 mm. Each trabecular bone sample was imaged using a high-resolution X-ray CT scanner (Phoenix Nanotom S, GE Sensing&Inspection Technologies GmbH, Wunstorf, Germany) equipped with a high-power nanofocus tube and a molybdenum target. Projection images on a CCD camera were obtained at 80 kV and 100 μA, with a resolution of 7.3 μm. A rotation of 0.18° was implemented between each image acquisition, providing a series of 2000 projection images. Phoenix Datosx2 software (v. 2.2, GE Sensing&Inspection Technologies GmbH, Wunstorf, Germany) was used to reconstruct a stack of two-dimensional sections from this series of projection images for each trabecular bone sample. A beam-hardening correction was applied during reconstruction using a method developed by GE Healthcare (GE Sensing and Inspection Technologies GmbH, 2010). Stacks were stored in TIFF files with indexed grayscale ranging from 0 to 255. The CT-Analyser software (v. 1.17.7.2, Bruker Micro CT, Kartuizersweg 3 B, Belgium) was used to calculate the bone volume fraction (BV/TV).

### 2.6. Uniaxial Mechanical Properties of Trabecular Bone

**Figure 2:**
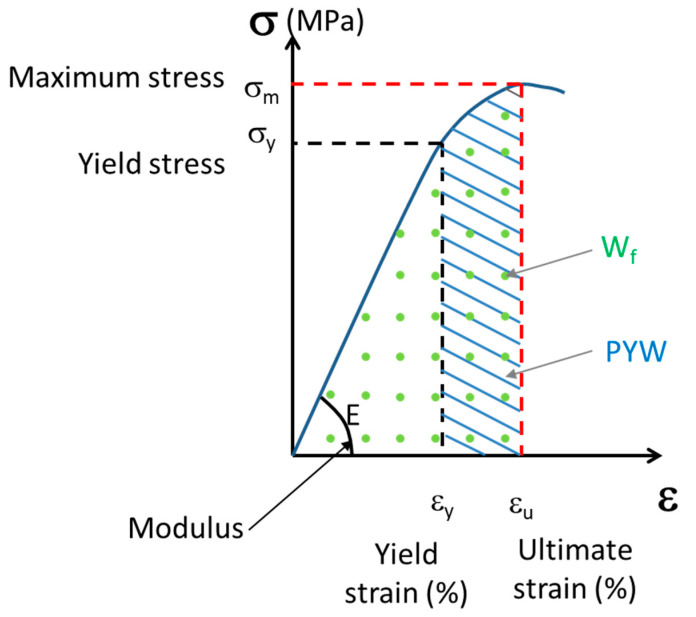
The schematic curve of uniaxial compression test of trabecular bone: E, elastic modulus; σ_y_, yield stress; ε_y_, yield strain; σ_m_, maximum stress; ε_u_, ultimate strain; W_f_, work of failure; and PYW, post yield work.

For each trabecular hydrated bone sample, a uniaxial compression test was performed along the cylinder at a constant displacement rate of 10^−2^ mm·s^−1^. The test comprised three steps: first, the sample was loaded to 10 N; next, the sample was unloaded; finally, it was loaded again to the maximum strength of the first plateau. Mechanical tests were performed using an INSTRON Electropulse 10000 (Instron World, Norwood, MA, USA) equipped with a DynacellTM 1 kN sensor. Strains were measured using a 25 mm gauge length extensometer attached to the end caps. The analyzed parameters (Figure 2) were the elastic modulus E, yield stress σ_y_, yield strain ε_y_, maximum stress (σ_m_) at the top of the plateau, ultimate strain (ε_u_) corresponding to the strain at the point of maximum stress, post-yield work (PYW), and failure work (W_f_).

### 2.7. Statistical Analysis

Continuous data are presented as the mean and standard deviation. All statistical tests were performed using the R software (v 4.2.2, R Foundation for Statistical Computing, Vienna, Austria). Given the small sample sizes, normality of distributions could not be guaranteed. Therefore, the statistical tests used in this study were non-parametric. Mann–Whitney U-tests were performed to assess statistical, qualitative differences between groups, with a significance level of 0.05.

## 3. Results

### 3.1. Effect of Osteogenesis Imperfecta on Skin Surface Morphology

**Figure 3:**
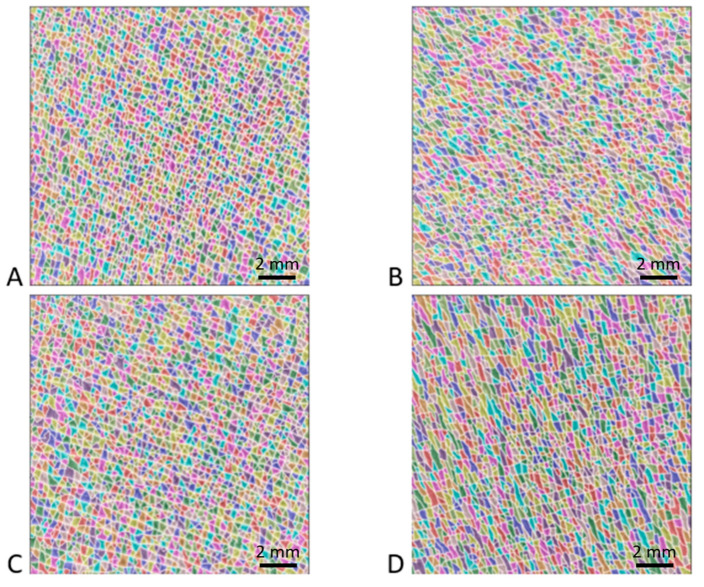
Results of watershed method applied to four high-resolution images (15 mm × 15 mm). Each color corresponds to a polygon of the patch surface network formed by the intersections of fine furrows and wrinkles. The first and second rows correspond to two patients aged 10 years and 15 years, respectively. The first and second columns correspond to non-pathological children and children suffering from OI, respectively. The different images correspond to (**A**) non-pathological 10-year-old patient, (**B**) OI 10-year-old patient, (**C**) non-pathological 15-year-old patient, and (**D**) OI 15-year-old patient.

Figure 3 shows typical images of the patch surface (15 mm × 15 mm) obtained for four patients: two non-pathological and OI patients aged 10 years and two non-pathological and OI patients aged 15 years. The high-resolution images processed using the watershed algorithm clearly show the shape of the polygons. In all the images analyzed, the polygons were fairly regular in shape and did not show significant elongation in any particular direction. Additionally, this treatment revealed a greater density of polygons in the non-pathological children than in the patients with OI. Similarly, these images suggest that the number of polygons decreased with age in both OI and non-pathological populations.

**Figure 4:**
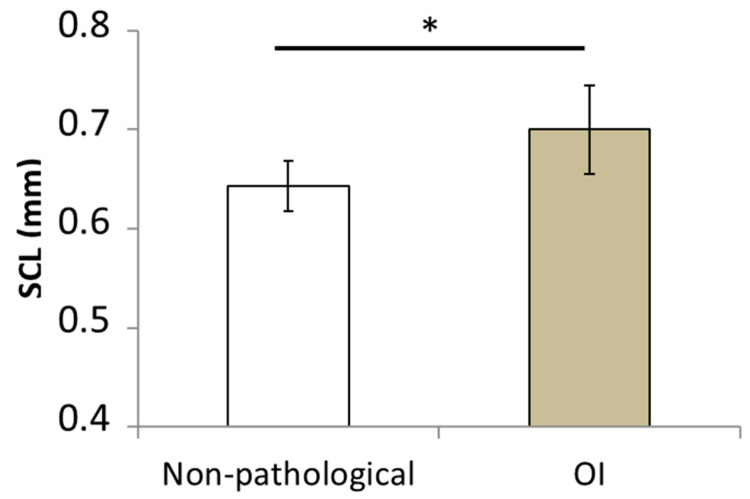
Mean values of skin characteristic length (SCL) computed for the group of healthy children and children suffering from osteogenesis imperfecta. (* *p*-value < 0.05).

The mean values for the areas of the polygons, the mean values for the perimeters of the polygons, and the SCL measurements are shown in Table 3 for all patients. The mean SCL value given in Figure 4 was significantly higher for the OI patients (0.7 ± 0.04 mm) than for the healthy children (0.65 ± 0.02 mm; *p* = 0.0015).

### 3.2. Effect of Aging

The mean values, standard deviations, and median values of the different mechanical parameters measured during the uniaxial compression test based on samples collected from 22 women over 60 years of age are listed in Table 4. This table also includes the mean values, standard deviation, and median values of the bone volume fractions obtained via X-ray microtomography, as well as the SCL values determined from the analysis of the skin patch surface. The SCL was significantly higher than that of the healthy children and children with OI (*p* = 1 × 10^−8^ and *p* = 1.35 × 10^−9^, respectively).

**Figure 5:**
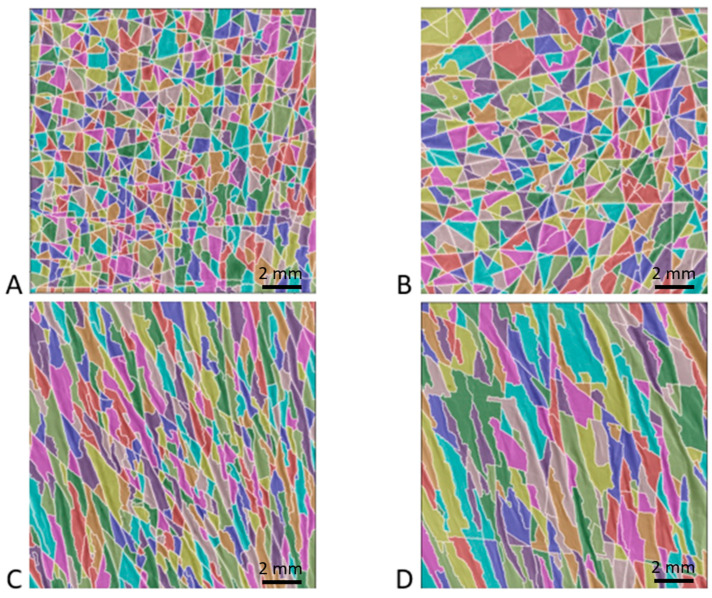
Example of four patches from senior women group processed using the watershed algorithm. Images (**A**,**B**) correspond to the typical morphology of a patient with a skin characteristic length below the median value of the elderly women group, whereas images (**C**,**D**) correspond to a value above this median value. (**A**) 79-year-old patient with an SCL of 1.05 mm; (**B**) 85-year-old patient with an SCL of 1.36 mm; (**C**) 61-year-old patient with an SCL of 1.85 mm; and (**D**) 85-year-old patient with an SCL of 2.65 mm.

All parameters showed strong inter-individual heterogeneity. In fact, this interindividual heterogeneity was observed in the four typical patch images shown in Figure 5. The polygon network obtained by processing the watershed algorithm was apparent. For instance, Figure 5A,B correspond to two patients whose SCL values were lower than the median value of the senior women group (1.05 and 1.36 mm, respectively). These images show, as in the case of the healthy children or OI patients, a distribution of larger isotropic polygons. Images 5C and 5D correspond to two patients whose SCL values were greater than the median values of the senior women, i.e., 1.85 and 2.65 mm, respectively. These images show the distribution of large, elongated polygons in a specific direction.

To quantify the visual inspection, Table 5 shows the average polygon area, perimeter, SCL, and REC for the 22 senior women, ranked from lowest SCL to highest.

**Figure 6:**
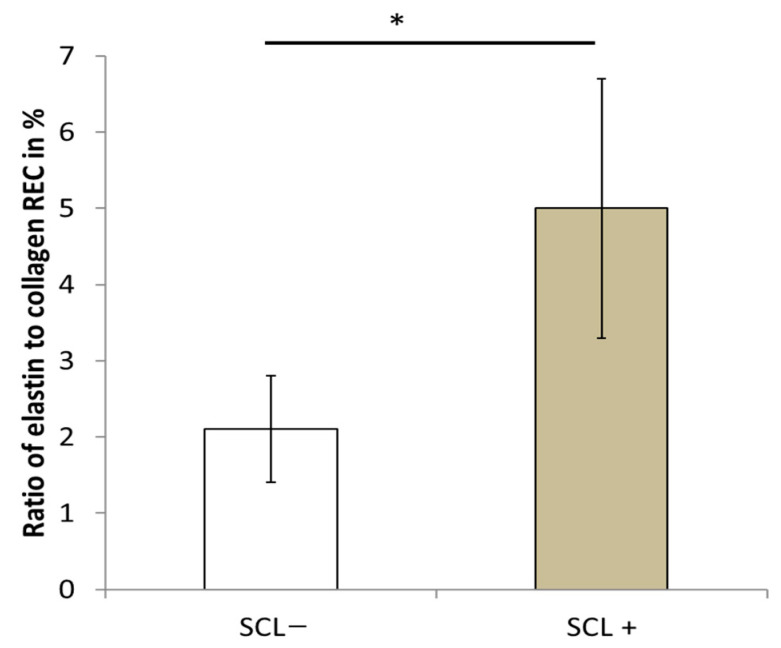
Ratio of elastin to collagen in the dermis according to the skin characteristics length. * *p* value < 0.05.

To investigate the relationship between SCL and the different parameters recorded in the present study, particularly the ratio of elastin to collagen, bone toughness, and ductility, the 22 patients were separated into two equal groups based on their SCL value. The first group denoted as SCL− defined in Table 5, included 11 women with an SCL value below the median value of 1.6 mm, whereas the second group, denoted as SCL+, defined in Table 5, included 11 women with an SCL value above the median value. The mean SCL values for the first and second groups were 1.25 ± 0.19 mm and 2.17 ± 0.37 mm, respectively. The relationship between SCL and REC measured in the dermis has already been shown in a previous study [42]. To confirm this observation, the elastin/collagen ratio (REC) values for the SCL− and SCL+ groups are shown in Figure 6 for the twenty-two older women. The SCL+ group had a significantly (*p* = 2.10^−5^) higher REC value of 5 ± 1.7% than the REC value of 2.1 ± 0.7% for the SCL− group. This confirms that a skin surface with more numerous and regular polygons has a lower REC. Since the amount of collagen decreases with age, and the amount of elastin increases with age, a low REC indicates a better quality dermis.

**Figure 7:**
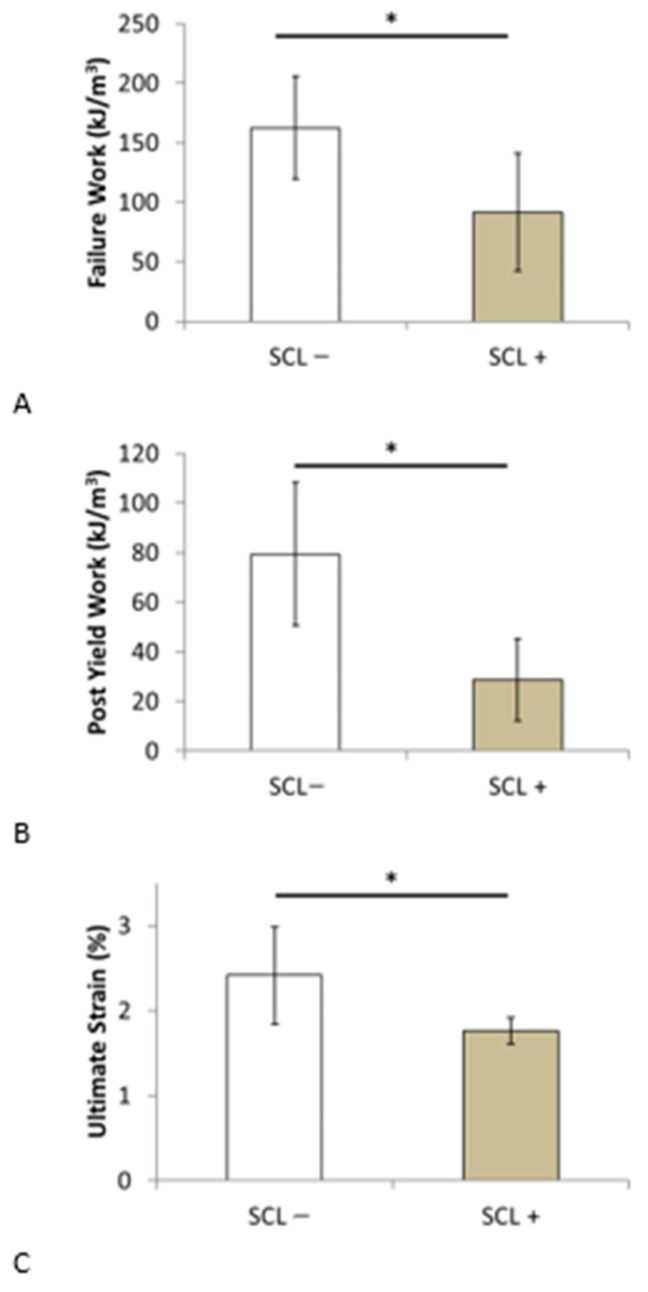
Comparison of (**A**) failure work, (**B**) post-yield work, and (**C**) ultimate strain between patients in senior women group with skin characteristic length lower (SCL−) and higher (SCL+) than the median value for this group. * *p* value < 0.05.

Table 6 shows the mean values ± standard deviation for the SCL− and SCL+ groups for the different parameters assessed in the present study and the probability value for evaluating the statistical differences between these two groups. The results showed that the mean demographic values, such as age, height, weight, and body mass index (BMI), were similar between the two groups. Regarding the bone parameters, the mechanical properties of the elastic domain (E, ε_y_, σ_y_), bone volume fraction, and maximum stress of the SCL− group indicated higher values than those of the SCL+ group, with a non-significant *p*-value. In addition, the values of the failure work and post-yield work of the SCL− group were significantly higher than those of the SCL+ group. This finding suggests a decrease in the trabecular bone’s ability to absorb energy with increasing SCL. Similarly, the ultimate strain value of the SCL− group was significantly higher than that of the SCL+ group, which is consistent with the decrease in ductility as the SCL increases (see Figure 7). The value of the post-yield work that does not consider the elastic aspect of the mechanical behavior is the one that decreases the most significantly with a divided value of approximately three.

## 4. Discussion

Type I collagen is the most important structural protein present in connective tissues. Its main role is to ensure the mechanical stability of these tissues and to provide a structural environment for cells and several biomolecules of the extracellular matrix. The conformation of the triple helix structure of this protein can be affected by a point mutation observed in certain genetic pathologies or by post-translational modifications such as AGEs. In contrast to the beneficial effects of enzymatic cross-links on the mineralization process and bone strength, AGEs decrease bone toughness, post-yield properties, and ductility [15,21,48]. Changes in the molecular structure of this protein are strongly associated with changes in the mechanical properties of these tissues, particularly in terms of strain and energy absorption features [11,16,30]. In the present study, two populations with increased bone fragility were investigated; in both cases, the SCL was shown to be significantly modified.

In fact, collagen aging in the collective unconscious is associated with the modification of the skin’s appearance, which results in wrinkles, color changes, or skin atrophy. Intrinsic skin aging, apart from environmental factors, such as the sun, results in a degradation in the microstructure of the dermis with age. The overall content of collagen is known to decline by approximately 1% per year [49], accompanied by an accumulation of partially degraded fibers [37,50]. The SCL used in the present study allowed us to assess microstructural degradation in the skin dermis. As expected from different skin studies [36,37,38,39,40,51], the SCL of the elderly women group was significantly higher than that of the children group.

Assuming that body collagen ages equally, the skin dermis and bone collagen should demonstrate the same kinetics [34,35]. As early as 60 years ago, McConkey et al. reported that the atrophy of the dorsal skin of the hands was qualitatively correlated with osteoporosis [52]. Subsequently, in vivo bone mineral density was compared with several skin parameters, such as collagen content, thickness, and elasticity [53,54,55,56], but only weak correlations were obtained. Recently, Haffer et al. [57] demonstrated a significant correlation between in vivo bone quality defined by AGEs and skin parameters measured using the ultrasound technique in lumbar fusion patients. All of these studies indicated that the relationship between skin and bone tissues should be investigated in terms of the quality of the organic phase of the bone matrix, which is associated with bone ductility, and its energy absorption capacity [10,16] instead of the bone mineral density, which is mainly related to bone stiffness.

The present study focuses on the potential relationship between skin morphology and bone biomechanical properties. Such a readily available relationship could complement and strengthen but not replace the current indicators used to predict fracture risk. In this context, the first population investigated in the present study was patients with mild osteogenesis imperfecta, which is a rare and systemic genetic collagen disease based on Sillence’s classification [58,59]. To the best of our knowledge, this is the first study where the skin surface morphology of this population with OI has been shown to be significantly different from that of a healthy population. OI pathology is primarily related to autosomal dominant mutation in genes encoding type I collagen (COL1A1 and COL1A2). Owing to its systemic nature, it affects all connective tissues, bones, skin, and teeth [60,61,62], and its main manifestation is bone fragility. Studies performed on human bone OI or murine models showed an increase in mineral content associated with a decrease in the organic matrix content [63,64,65,66]. From a mechanical perspective, the study of Grafe et al. [43] showed that toughness and post-yield displacement reduced by a factor of five in the OI mouse model compared with the wild type, whereas the elastic modulus and elastic displacement were similar between OI and wild-type mice. The study of Carrriero et al. [44], which also showed a significant decrease in toughness, attributed this decrease to collagen-altered fibril packing and intermolecular cross-linking with an increase in AGEs. All these results confirm that bone mineral density and collagen quality impose separate effects on the mechanical properties; collagen quality and, in particular, AGEs mainly affect the toughness and ductility of bone tissue. This finding is consistent with the finding that the SCL of the OI group was significantly higher than that of the healthy group.

The mechanical properties of trabecular bone depend significantly on the sampling site, i.e., the femur, tibia, or vertebrae, as well as the age and sex of the individual [67]. Therefore, obtaining an exact value for the different mechanical parameters measured in the present study was difficult. In the present study, trabecular bone cores were always taken from the femoral head in the same anatomical position and direction. This was done to avoid variations in mechanical results from one patient to another due to the experimental procedure. However, the average values of the different experimental bone mechanical parameters were of the same order of magnitude as those reported in the literature [4,68,69,70]. A decrease in bone toughness with aging has been reported [71,72]. Non-enzymatic glycation has been suggested to be a major contributor to the deterioration in the bone organic matrix, thus resulting in a decrease in toughness and post-yield properties [15,19,30,48,73]. In the present study, we hypothesized that the SCL is a marker of collagen quality in bone tissue. A group of 22 elderly women was segregated into two subgroups based on the SCL value of each individual. The two groups showed no significant differences in age, bone volume fraction (BV/TV), or elastic mechanical parameters. However, the mean values of bone toughness and ductility were significantly lower in the group with a higher SCL.

Vörös et al. [74] have already investigated age-related changes in the area and perimeter of polygons measured on the skin surface of the forearm in 190 patients, including 39 women over 60, from a general population. These authors show that in women over the age of 60, the results are very scattered. In this age group, the mean polygon areas varied from 0.82 mm^2^ to 0.2 mm^2^. These values are slightly lower than those obtained in the present study, which ranged from 1.3 mm^2^ to 0.16 mm^2^. Similarly, in the study by Voros et al., the perimeters ranged from 1.67 mm to 3.7 mm. These values are lower than in the present study, which ranged from 2 mm to 6.6 mm. These differences may be a result of the measurement technique, which has since been improved with the development of computer-aided tools. However, in the same study, Vörös et al. reported a mean polygon perimeter length of 1.38 mm at the age of 15 years. This value is very close to the mean value of 1.4 mm that was obtained in the present study at the same age for the non-pathological group. Therefore, this finding implies that the elderly population in the present study has a larger polygon perimeter than the general population and, consequently, a larger SCL, suggesting lower bone ductility and toughness. This finding is consistent with the characteristics of the study population who have experienced simple falls and are, therefore, more fragile than the general population. Hence, the SCL provides in vivo access to deteriorated bone toughness.

Some limitations of the present study must be addressed. Our results were obtained from two clinical studies performed on specific populations with increased bone fragility: children with OI and women over the age of 60 years. Particularly for older women, it is important to note that the division into SCL− and SCL+ is within a population that sustained a hip fracture after a simple fall. Therefore, the separation into two groups makes it possible to establish the relationship between SCL and the biomechanical properties of bone tissue. However, it does not allow us to conclude whether this indicator is predictive of fracture risk. The use of SCL in current clinical practice in older women needs to be further investigated to assess its ability to improve the predictive indices of fracture risk. In addition, elderly male patients were excluded from the analysis. Bone biopsies from elderly women were obtained from the femoral head, and other anatomic sites, such as the vertebrae, should be investigated. In particular, the area near the femoral neck, which is the main site of femoral fractures, should be studied. It is also important to note that the biomechanical characterization of bone tissue in older women was performed using a compression test on a bone core to obtain a representative bone volume. However, from a biological point of view, the compression test is certainly not the most appropriate to study the relationship between collagen and the mechanical properties of bone tissue. Furthermore, the relationship between SCL and the post-yield mechanical properties of bone highlighted in the present study suggests biological deterioration of the bone matrix. Based on various studies, the biological mechanism underlying this relationship might be related to the accumulation of AGEs [14,48,57]. However, the present study did not measure collagen quality using micro-spectroscopic techniques. Although the relationship between SCL and some bone biomechanical properties has been demonstrated, from a biological point of view, the relationship between SCL and collagen quality, in particular AGE accumulation in bone tissue and dermis [75], is unknown and requires further investigation.

A relationship between the SCL based on the in vivo measurement of skin surface patterns and the mechanical properties of bone was demonstrated. The SCL can reveal systemic collagen deficiency (osteogenesis imperfecta). In addition, bone degradation with age resulted in a significant increase in the SCL. Furthermore, in women aged over 60 years, larger SCLs resulted in decreased bone toughness and ductility. The in vivo measurement of SCL, which assesses the quality of bone collagen in vivo, may be incorporated into future clinical practice to improve the prediction of bone fracture risk. In particular, the effect of patient age, especially the relationship between bone collagen quality and SCL for children, will be investigated in the near future. Owing to its non-invasive nature and near-immediate results, SCL measurement should be incorporated into routine patient-specific practice to monitor changes in bone matrix quality.

## Figures and Tables

**Table 1:** Patients’ characteristics of OI and non-pathological groups were analyzed in the present study.

OI N°	Sex	Age (Year)	Height (cm)	Weight (kg)	Non-Pathological N°	Sex	Age (Year)	Height (cm)	Weight (kg)
1	M	8	119	24	1	F	10	134	23
2	F	10	106	20	2	M	13	158	46
3	M	12	147	52	3	M	15	175	60
4	M	12	147	38	4	M	15	181	80
5	F	13	148	37	5	M	15	172	60
6	M	13	159	43	6	F	15	170	60
7	M	13	125	30	7	F	17	163	53
8	F	14	152	35	8	F	17	170	52
9	M	14	155	55	9	M	17	180	75
10	M	14	152	34	10	M	17	182	78
11	F	15	159	59	11	F	20	170	50
12	F	16	127	31					
13	M	18	130	38					
Mean value		13.3 (±2.4)	140 (±16)	38.2 (±11)			15.5 (±2.4)	168 (±13)	57.9 (±15.6)

**Table 2:** Characteristics of the senior women group analyzed in the present study.

	Age (Years Old)	Weight (kg)	Height (in cm)	BMI
1	61	65	165	23.8
2	95	55	160	21.45
3	68	64	165	23.5
4	76	69	168	24.4
5	89	74	160	28.9
6	85	59	164	27.7
7	79	52	162	19.8
8	96	40	150	17.8
9	87	56	156	23.
10	74	64	165	23.5
11	94	49	162	18.7
12	80	70	165	25.7
13	89	50	160	19.5
14	75	60	171	20.5
15	89	55	147	25.5
16	91	46	158	18.4
17	85	60	169	21.
18	81	70	160	27.3
19	82	54	160	21.1
20	73	61	166	22.1
21	67	65	160	25.4
22	86	73	165	26.8
Mean value	81.9 (±9.3)	59.6 (±8.8)	161 (±.6.4)	23 (±3.1)

**Table 3:** Skin characteristics of OI and non-pathological groups analyzed in the present study.

OI N°	Mean Polygon Area (mm^2^)	Mean Perimeter Length (mm)	SCL (mm)	Non-Pathological N°	Mean Polygon Area (mm^2^)	Mean Perimeter Length (mm)	SCL (mm)
1	0.092	1.42	0.65	1	0.078	1.31	0.60
2	0.086	1.4	0.64	2	0.079	1.31	0.6
3	0.11	1.71	0.75	3	0.085	1.37	0.63
4	0.11	1.54	0.71	4	0.085	1.44	0.65
5	0.12	1.64	0.75	5	0.093	1.44	0.66
6	0.096	1.45	0.67	6	0.09	1.39	0.64
7	0.107	1.52	0.7	7	0.1	1.51	0.69
8	0.12	1.67	0.76	8	0.09	1.43	0.65
9	0.093	1.47	0.67	9	0.098	1.48	0.68
10	0.1	1.44	0.67	10	0.095	1.44	0.66
11	0.11	1.59	0.72	11	0.092	1.43	0.66
12	0.135	1.73					
13	0.106	1.53					
Mean value	0.11 (±0.01)	1.57 (±0.1)	0.71 (±0.04)		0.09 (±0.007)	1.4 (±0.06)	0.65 (±0.02)

**Table 4:** Mean values, standard deviation, and median values of bone and skin parameters analyzed in the present study: Elastic modulus, E; yield stress, σ_y_; yield strain, ε_y_; maximum stress, σ_m_; ultimate strain, ε_u_; post yield work, PYW; failure work, W_f_; bone volume fraction, BV/TV; and skin characteristic length, SCL.

	Mean Value	Standard Deviation	Median Value
E (MPa)	710	322	678
σ_y_ (MPa)	9.43	4.3	9.4
ε_y_ (%)	1.51	0.31	1.44
σ_m_ (MPa)	10.2	4.59	10.8
ε_u_ (%)	2.08	0.53	1.88
PYW (kJ/m^3^)	54	34.6	53.6
W_f_ (kJ/m^3^)	127	58.3	133
BV/TV (%)	23.75	6.4	24.6
SCL (mm)	1.71	0.55	1.6

**Table 5:** Average polygon area, perimeter, SCL, and REC for the 22 senior women ranked from lowest SCL to highest.

N°	Mean Polygon Area (mm^2^)	Mean Perimeter Length (mm)	SCL (mm)	REC (%)
Group SCL−				
21	0.16	2.0	0.90	1.25
7	0.22	2.3	1.05	2.30
15	0.21	2.4	1.06	1.45
20	0.24	2.4	1.10	2.19
19	0.27	2.6	1.18	2.21
16	0.32	2.8	1.29	1.22
22	0.34	3.0	1.35	2.70
17	0.37	3.0	1.36	0,60
2	0.36	3.0	1.37	3.00
18	0.39	3.3	1.46	1.40
12	0.47	3.5	1.56	3.08
Group SCL+				
14	0.45	3.7	1.62	2.66
10	0.43	4.0	1.66	3.87
1	0.53	4.4	1.84	3.63
4	0.64	4.1	1.85	3.46
11	0.74	5.2	2.17	4.36
3	0.61	5.6	2.19	5.00
5	0.87	5.2	2.25	4.94
13	0.96	5.3	2.31	5.18
9	0.74	6.6	2.52	5.56
6	1.04	6.5	2.65	8.43
8	1.39	6.6	2.8	7.85

**Table 6:** Mean values and standard deviation of demographic data (age, weight, height, BMI) and bone parameters (Elastic modulus, E; yield stress, σ_y_; yield strain, ε_y_; maximum stress, σ_m_; ultimate strain, ε_u_; post yield work, PYW; failure work, W_f_; bone volume fraction BV/TV) between patients in senior women group with skin characteristic length lower (SCL−) and higher (SCL+) than the median value for this group.

	SCL− (n = 11)	SCL+ (n = 11)	*p* Value
Demographic Data			
Age (Years)	82.54 ± 7.6	81.27 ± 10.7	0.97
Height (cm)	161.1 ± 5.5	160.7 ± 7.2	0.97
Weight (kg)	60.1 ± 8.2	59.1 ± 9.3	0.89
BMI (kg/m^2^)	23.15 ± 2.9	22.85 ± 3.4	0.79
Bone Parameters			
E (MPa)	729 ± 277.2	691 ± 360.5	0.56
σ_y_ (MPa)	10.52 ± 3.6	8.34 ± 4.6	0.29
ε_y_ (%)	1.61 ± 0.4	1.41 ± 0.14	0.49
σ_m_ (MPa)	11.34 ± 3.8	9.05 ± 4.89	0.37
ε_u_ (%)	2.41 ± 0.57	1.75 ± 0.15	<0.05
PYW (kJ/m^3^)	79.3 ± 28.9	28.6 ± 16.6	<0.05
W_f_ (kJ/m^3^)	162.6 ± 42.9	91.6 ± 49.4	<0.05
BV/TV (%)	25.9 ± 5.4	21.5 ± 6.5	0.27

## Data Availability

The data presented in this study are available on request from the corresponding author. The data are not publicly available due to ethical reasons.
